# The Critical Role of Mental Health Specialists in Organ Transplantation Teams: An ELPAT Position Statement

**DOI:** 10.3389/ti.2026.15605

**Published:** 2026-05-08

**Authors:** Inês Mega, Manuela Almeida, Angela Buchholz, Rosie Heape, Martin Kumnig, Emma K. Massey, Mariel Nöhre, Anne Skorzewski, Hannah Maple

**Affiliations:** 1 Hepato-Biliary-Pancreatic and Transplantation Centre, Curry Cabral Hospital, ULS São José, NOVA Medical School, Lisbon, Portugal; 2 Department of Nephrology, Centro Hospitalar Universitário de Santo António, Unidade Local de Saúde de Santo António (ULSdSA), Oporto, Portugal; 3 Department of Medical Psychology, University Medical Center Hamburg-Eppendorf, Hamburg, Germany; 4 Faculty of Life Sciences and Medicine, King’s College London, London, United Kingdom; 5 Department of Psychiatry, Psychotherapy, Psychosomatics and Medical Psychology, Center for Advanced Psychology in Plastic and Transplant Surgery, Medical University of Innsbruck, Innsbruck, Austria; 6 Section of Nephrology and Transplantation, Department of Internal Medicine, Erasmus MC Transplant Institute, Rotterdam, Netherlands; 7 Department of Psychosomatic Medicine and Psychotherapy, Hannover Medical School, Hannover, Germany; 8 University of Utrecht, Utrecht, Netherlands; 9 Department of Renal Transplantation, Guy’s and St Thomas’ NHS Foundation Trust, London, United Kingdom

**Keywords:** adherence, mental health, organ transplantation, psychosocial care, psychosocial wellbeing

## Abstract

Transplant patients face complex medical and psychosocial challenges that require multidisciplinary protocols and care plans. Despite this, mental health support remains varied across centers and countries, leading to unmet mental health needs. The psychological impact of transplantation is evident before and after transplantation. Transplant patients at any stage face several challenges. Non-adherence to medications is common and can be a result of the psychological burden. A transplant mental health service could address these problems and, consequently, help improve quality of life and adherence, reduce complications, and prolong graft survival. We believe this might be beneficial from an economic perspective as well, even though further research is needed. We propose a comprehensive approach based on the biopsychosocial care model for integrating specialized mental health professionals into transplant teams across all phases of care. We call upon transplant centers, medical societies, insurance providers, and policymakers to recognize the importance of mental health expertise in transplantation.

## Introduction

Organ transplantation represents one of modern medicine’s most remarkable achievements, offering life-saving interventions for patients with end-stage organ failure. It is a distinctive field of healthcare, best understood as an existential turning point with psychological, emotional, and social implications [1]. It relies upon the expertise of several individuals who have collectively enabled its rapid expansion through advancements in science, medicine, and surgical techniques. Consequently, transplant candidates and recipients are often confronted with complex medical and psychosocial challenges [2–5].

With the increasing complexity of organ transplantation, the psychosocial aspects accompanying transplant candidates have also changed. The relationship between physical and psychosocial health is now better understood, and therefore, patients are accepting and asking for mental health support as part of their transplant journey [6–8]. This need is often unmet, with financial investment by transplant programs remaining sporadic and underfunded.

This position paper is written on behalf of the Psychological Care working group, which forms part of ELPAT; the Ethical, Legal and Psychological Aspects of Transplantation section of the European Society for Organ Transplantation. It provides both a rationale and an evidence base to support the critical role of mental health professionals (MHPs) within organ transplantation and its integration into standardized care in the adult transplant population.

## Definition of a Mental Health Professional

The role of MHPs in the field of transplantation medicine is to identify psychological stress and mental disorders, to communicate psychosocial aspects concerning the patient within the interdisciplinary transplant team, to assess their influence, and to apply appropriate interventions accordingly. To perform this role effectively, several prerequisites are essential. First, appropriate qualifications in the diagnosis and treatment of mental disorders are required. Although there are some differences between different European countries - and major differences compared to the United States - regarding the training and qualifications of the various professional groups, this definition includes the following professions: clinical psychologists, psychotherapists or specialists in psychiatry or psychosomatic medicine.

In addition, sufficient theoretical knowledge and clinical experience in transplantation medicine are required [9].

Other professions, such as nurses or social workers, also play an important role in caring for this patient group. Tasks like providing education, implementing interventions to improve adherence, or conducting screenings to detect mental disorders can and should also be carried out by them. However, if pronounced psychological distress is present and the criteria for a mental disorder are met, the involvement of an MHP appears indispensable.

## The Current State of Psychosocial Assessment and Intervention Before Transplantation

The psychosocial support of transplant candidates and recipients is inconsistent across the world, varying from nothing at all to a comprehensive one-to-one evaluation by a fully trained MHP and a subsequent psychological intervention. There are different recommendations and clinical guidelines from experts in the field for different organs and/or countries [2, 3, 9–11]. Guidelines comprise recommendations regarding assessment of mental health problems by an MHP [2, 3, 9–11], usage of psychosocial evaluation tools [2, 3, 9, 11], offering interventions for patients with mental disorders [3, 9–11], and inclusion of an MHP in a multidisciplinary collaborative team [2, 3, 9–11].

Psychosocial assessment tools have been developed to supplement the evaluation of solid organ transplant candidates, with some predictors of psychosocial and somatic outcomes starting to emerge [5–18]. Tools (such as the SIPAT [19], TERS [20] and PACT [21]) address domains that are relevant for all transplant populations. These include a history of psychopathology, issues with family support, alcohol and/or substance use disorder, knowledge about transplantation, and a history of non-adherence. Other domains include relationships with living donors, acceptance of an organ, and concerns around bodily integration [22].

Whilst assessment tools help transplant professionals conduct a psychosocial evaluation, they are of limited use by those who are not trained to diagnose mental disorders, or who lack understanding of the unique psychological landscape of transplantation. Conversely, MHPs with specialized training and expertise need an understanding of transplant procedures, basic immunology, medication regimes, and their side effects [9, 23, 24]. The assessment of a transplant patient by an MHP with this level of knowledge is likely to be superior to that of a general mental health clinician with no expertise within transplantation and a transplant professional with no expertise in mental health.

Even though there is some evidence that psychosocial interventions before and after organ transplantation can be effective in improving mental wellbeing, treatment adherence, and quality of life [1, 9, 13, 14], many transplant programs lack a dedicated MHP at any stage of the transplant process.

## Pre-Transplant Care and the Psychological Burden of Waiting

Waiting for an organ transplant creates extraordinary psychological strain that current care models inadequately address. According to the literature [1, 25–29] and our own clinical experiences, waitlisted patients are likely to encounter:

- Existential uncertainty: Living in a prolonged state of limbo between hope and despair while facing mortality daily.
- Anticipatory anxiety: Constant hypervigilance and the burden of perpetual readiness for “the call”.
- Identity disruption: Fundamental challenges to self-concept as illness progresses and dependency increases.
- Social isolation: Withdrawal from normal activities and relationships due to physical limitations and the unpredictability of the waiting process.

Research demonstrates that relevant proportions of patients on transplant waiting lists experience clinically significant depression and anxiety (Table 1). Depression, in particular, is associated with decreased survival rates after transplantation [9, 12, 30, 31, 32]. Furthermore, pre-transplant psychological distress predicts post-transplant non-adherence and inferior clinical outcomes [33, 34].

**TABLE 1:** Frequencies of anxiety and depression before and after organ transplantation (adapted from [47]).

Mental disorder	Prevalence before transplantation	Prevalence after transplantation
Depressive disorders		
Heart	24%–50% [35–38]	17%–38% [39–41]
Liver	23%–60% [42–44]	30%–40% [43, 45]
Lung	12%–25% [30, 46, 48]	16%–37% [46, 49]
Kidney	37%–42% [50–53]	13%–33% [51, 52, 54]
Anxiety disorders		
Heart	8%–16% [36, 37]	17%–53% [36]
Liver	14%–20% [42, 44]	10%–26% [55]
Lung	40%–58% [48]	20%–60% [56]
Kidney	32% [57]	20% [57]

## Post-Transplantation Psychological Challenges

Receiving an organ transplant does not resolve the underlying psychological distress but transforms it. Post-transplant patients face unique challenges, which may include:

- Survivor’s guilt: Complex emotions about receiving an organ, particularly from deceased donors [58].
- Identity integration: Psychological adaptation to having another’s organ within one’s body, with patients having to renegotiate their body identity and sense of self while incorporating a “foreign” organ [1, 22, 59, 60].
- Fear of rejection or disease recurrence viewed as a persistent anxiety about organ failure despite medical reassurance [58].
- Adaptation to the “sick role” paradox: Navigating life as simultaneously “cured” yet requiring lifelong medical management [61].
- Medication burden: Managing complex immunosuppressive regimens with significant side effects [58].
- Relationship with a living donor: expressing gratitude, guilt, and navigating potential changes in a relationship [62], while experiencing a sense of indebtedness difficult to elaborate [1].

Depression is frequent after organ transplantation (Table 1) and is associated with higher morbidity and mortality after organ transplantation [8, 31, 34]. In the meta-analysis by Dew et al. [12], the impact of post-transplant depression on mortality was more substantial compared to pre-transplant depression. Several mechanisms may explain the association between depression and poor graft outcomes. Depression often leads to negative health behaviors such as non-adherence to treatment and appointments, which are major risk factors for graft failure [63] as described below. Depression is also seen as a modifiable risk factor and may be improved by early detection and treatment. A study in liver transplant recipients [31] demonstrated that those with adequately treated depressive disorders had improved survival rates, whereas untreated depression was identified as a risk factor for long-term mortality.

Additionally, a relationship between depression and substance use, inadequate diet, and exercise has been reported [64]. Regarding the prevalence of anxiety and depression, several studies are available (Table 1). The prevalence of PTBS following organ transplantation ranges 1%–16% (clinician-ascertained) and 0%–46% (self-report instruments) [50]. There are only a few studies on transplant patients that have diagnosed mental disorders according to the ICD- or DSM-classification [12]. Most studies have used standardized self-report instruments with validated cut-offs to detect mental disorders. As self-report instruments might overestimate specifically the prevalence of depressive symptoms due to an overlap of symptoms between physical and mental disorders, a secure diagnosis based on established diagnostic criteria performed by an MHP is necessary to provide adequate treatment recommendations [47].

## Non-Adherence

Adherence is a complex construct within which the WHO proposed five dimensions, each clustering different variables [65]. To positively influence adherence, detecting non-adherence and understanding individual barriers is crucial. Although necessary, adherence screening, often performed by transplant nurses or coordinators, is not standard practice at all European transplant centers. Depending on the difficulties identified, e.g., forgetfulness, medication side effects, being overwhelmed by the complexity of the medication regimen, performing tutorials, and developing individualized strategies are effective intervention methods. However, non-adherence to treatment can result from the psychological burden prominent in transplant patients [13, 65]. One of the most compelling arguments for the integration of MHPs into transplant teams lies in the undeniable relationship between psychological wellbeing and adherence to different components of post-transplantation care. It has been shown that:

- Patients with depression have a higher risk of non-adherence to immunosuppressive medications [60, 66].
- Anxiety can paradoxically lead to avoidance of medical environments and medication non-adherence [67].
- Executive functioning impairments related to psychological distress directly affect an individual’s ability to follow complex medication regimens [68].
- Multimodal interventions, including psychological elements, are able to improve adherence [10, 69, 70].

## Economic Implications

Whilst there is limited direct data on the economic benefits of embedding MHPs within transplant teams, the financial benefits of transplantation and maximizing graft outcomes are undeniable compared to alternatives, such as dialysis. Therefore, we can infer substantial economic advantages from reducing psychosocial morbidity within the pre- or post-transplant population due to the impact of patients on eligibility for transplant listing and physical outcomes.

We would like to present some calculation examples based on available data: The highest paid clinical psychologists in the UK earn around 80,000 € per annum [71], and whilst such a professional would incur other costs within the system (i.e., a clinical work area, office space etc.), these are trivial when compared to the costs of dialysis, which are around 40,000 € each year per patient. It follows that if one psychologist can prevent just two people from needing dialysis (either by helping them enter a suitable condition for transplantation or by prolonging graft survival) for 12 months, then their salary has paid for itself.

Further economic benefits could be obtained through additional quality-adjusted life years and reduced non-adherence-related pathologies, such as antibody-mediated rejection (ABMR) [72], which costs at least $30,000 per episode [73]. In addition to the treatment costs of ABMR, there are further costs if the graft fails, including rehospitalization and retransplantation. The costs are further amplified if the person develops antibodies, making them more difficult to transplant again. The human cost of preventable organ rejection cannot be overstated. The integration of an MHP into transplantation services is therefore consistent from a purely economic perspective. Further research is needed to better quantify this economic benefit.

## Ethical Considerations

Beyond outcome measurements, ethical principles compel us to address the psychological dimensions of transplantation [74]:

- Respect for autonomy: Psychological support enhances patients’ capacity for informed decision-making.
- Beneficence: Addressing psychological suffering is a direct good, not merely an adjunct to medical care.
- Non-maleficence: Failing to address psychological needs constitutes a form of harm in itself.
- Justice: Equal access to psychological support ensures equitable distribution of complete transplant care.

The ethical issues of transplantation need to be discussed with candidates, balancing the potential risks against anticipated benefits. Where psychosocial issues are prevalent, a dedicated MHP is essential to facilitate complex ethical considerations with patients.

## Comprehensive Implementation Model

A successful transplant is built upon the foundations of a multi-staged, multi-disciplinary evaluation of the candidate; an integral component is the psychosocial evaluation. After careful selection of those most likely to benefit, interventions designed to minimize emotional distress and facilitate integration and adaptation need to be tailored to transplant patients based on their psychosocial assets, with psychosocial support enabling patients to successfully adjust to life after a transplantation.

Based on the biopsychosocial model [75–77], we advocate for a comprehensive model, integrating specialized MHPs into transplant teams across all phases of care, working alongside other transplant professionals and comprising the following:

### Pre-Transplant Phase

- Standardized psychological assessment for all transplant candidates using validated instruments.
- Psychotherapeutic preparation protocols, including stress management techniques tailored to individual needs.
- Targeted interventions for high-risk patients based on identified vulnerabilities.
- Family system assessment and support to strengthen the patient’s social network.
- Addressing potential barriers to adherence *a priori*.

### Waiting Period

- Regular psychological monitoring with validated instruments to track changes in mental health status.
- Group therapy opportunities with professional guidance.
- Support measures in self-help/peer support.
- A crisis intervention protocol for acute psychological distress during the uncertain waiting phase.
- Telepsychology options for geographically distant patients.
- Development of coping strategies specifically tailored to the challenges of waiting.

### Post-Transplant Phase

- Post-operative psychological support to address surgical recovery stressors.
- Structured transition program from inpatient to outpatient care.
- Medication adherence screening for early identification of non-adherence.
- Establishment of a medication adherence enhancement protocol based on evidence-based behavioral approaches.
- Long-term adjustment counseling at key milestones to address evolving psychological needs.
- Early intervention for emerging psychological symptoms [1, 9, 12, 31].
- Intervention protocol for psychological impact of graft failure.

### Interprofessional Teamwork

- Weekly multidisciplinary team meetings with equal voice for MHPs [76].
- Joint medical-psychological rounds to facilitate integrated care [76].
- Cross-training for all team members in basic psychological support principles [76].
- Implementing screening for symptoms of mental disorders [9].
- Shared electronic medical record documentation for seamless communication [76].
- Regular multidisciplinary case reviews of complex patients incorporating both medical and psychosocial aspects [76].

### Specialized Training

Requirements may include:

- Advanced training in psycho-nephrology, psycho-hepatology, or other organ-specific psychological subspecialties.
- Certification in transplantation psychology through established programs.
- Regular participation in transplant-specific continued education.
- Supervised practice within transplant settings before independent practice.
- Ongoing professional development in both transplant medicine and psychological interventions.

## Barriers and Solutions

Several barriers obstruct implementing a comprehensive model. The main constraint is financial, as budgeting for mental health services is difficult in the current economic climate. As described above, an economic analysis might conclude that a comprehensive model can save money by reducing readmission rates and complications, and by prolonging graft and patient survival. Subsequently, transplant professionals should advocate for payment models that routinely include psychological services.

Additionally, there is a shortage of specialized MHPs. Only a few mental health trainees are being exposed to transplantation, and there is a lack of standardized training. A solution for this is to develop a standardized curriculum within specialist training programs, including certification pathways and credentialing standards, partnerships with established transplant and academic institutions and societies, and incentives for specialization in transplant psychology.

Stigma surrounding mental healthcare may exist in both patients and professionals. Patients may fear being removed from transplant lists due to psychological issues, so clear communication about the supportive rather than exclusionary function of psychological assessment and about the benefits of psychological care is crucial. Likewise, there may be resistance within professional groups where traditional perspectives may marginalize mental health input and those who provide it. Solutions to this may include changes to management approaches and leadership engagement, evidence-based advocacy, and shared success stories from centers with integrated models. Normalization of psychological assessment may reduce stigma, both for patients and transplant professionals.

## Conclusion

This paper provides both a rationale and evidence base in support of psychosocial care becoming a standardized and routine part of transplantation. We presented evidence that organ transplantation is not merely a biomedical procedure, but a profound psychological journey that fundamentally transforms patients’ lives. To achieve the best outcome, which is to help and not to harm, a multidisciplinary team including a specialized MHP is key [76]. By addressing mental health in transplant care and helping patients adjust to the complexities of the process, graft longevity and patient survival might be enhanced. Additionally, the MHP can offer support and training to other members of the multidisciplinary transplant team, helping to improve effective communication, motivation, and a non-judgmental approach to taboo subjects.

There is a need for further research on evaluating mental health interventions in transplant patients and on measuring the economic value of these interventions on the long-term course.

As a group of MHPs, educationalists, ethicists, and clinicians specializing in transplantation, we propose that dedicated MHPs should be integral, mandated members of all multidisciplinary organ transplant teams. We call upon transplant centers, medical societies, insurance providers, and policymakers to recognize that comprehensive transplant care must include mental health expertise from professionals specifically trained in transplantation as members of multidisciplinary teams. The unique psychological challenges of transplantation require specialized knowledge that general mental health training does not provide. Just as we would not expect a general surgeon to perform a liver transplant, we should not expect general mental health clinicians to address the complex psychological dimensions of transplantation.

Maximizing the survival and quality of life of transplant patients is the primary goal of solid organ transplant programs. As the field advances technologically, we must ensure that our care models advance equally in addressing the psychological dimensions of transplantation. Only then can we claim to truly honor the extraordinary gift of donated organs and the lives they have the potential to transform.

## Data Availability

The original contributions presented in the study are included in the article/supplementary material, further inquiries can be directed to the corresponding author.
